# Case Report: CD34-Negative, S100-Positive Spindle Cell Tumor With a *MYH10-RET* Fusion

**DOI:** 10.1155/crip/9442676

**Published:** 2025-08-19

**Authors:** Yifei Wang, Xiaowei Li, Chunfang Yao, Zhou Zhou, Wendong Liu, Hengli Ni

**Affiliations:** ^1^Department of Pathology, Children's Hospital of Soochow University, Soochow University, Suzhou, China; ^2^Department of Orthopedic Surgery, Children's Hospital of Soochow University, Soochow University, Suzhou, China

**Keywords:** case report, MYH10, NTRK, RET, soft tissue tumor

## Abstract

With the application of next-generation sequencing (NGS) in soft tissue tumors, *NTRK* gene rearrangement is becoming known to pathologists as a molecular hallmark of spindle cell tumors, and other spindle cell tumors with related kinases are being reported. However, cases of *RET* rearranged spindle cell tumors not associated with *NTRK* rearrangements are rarely reported. Here, we describe a case of *RET* rearranged spindle cell tumor in a 3-year-old girl who presented with swelling and pain in the arm. Histologically, the tumor consisted of a fascicular arrangement of monomorphic spindle cells infiltrating and growing in adipose and muscle tissue with visible mitotic activity. Immunohistochemistry showed spindle cells negative for CD34, positive for S100 protein, and focal staining for Pan-TRK. *MYH10-RET* fusion was identified by NGS. Fluorescence in situ hybridization (FISH) analysis confirmed the *RET* gene rearrangement but did not detect the *NTRK1/2/3* gene rearrangement. In conclusion, we describe a rare case of CD34-negative, S100-positive spindle cell tumor with *MYH10-RET* fusion.

## 1. Introduction

*NTRK* and other related kinase-rearranged spindle cell neoplasms are new types of pathohistology defined by molecular features in WHO classification of soft tissue tumors 2020 [1, 2]. *RET* rearranged spindle cell tumors are one of the less reported types. *RET* fusions occur in fewer than 1% of soft tissue tumor cases, and *NTRK* fusions occur in fewer than 1% of adult and pediatric solid tumors [3, 4]. According to the published literature, *RET* rearranged spindle cell tumors exhibit variable morphologic and histologic grading from low to high grade, including lipofibromatosis-like neural tumor (LPF-NT), such as malignant peripheral nerve sheath tumor (MPNST) or infantile fibrosarcoma (IFS) [5]. *NTRK* rearranged spindle cell neoplasm has been studied more frequently, and the tumor occurs in infants, young children, adolescents, and less frequently in adults [6]. Immunohistochemistry (IHC) often shows dual expression of S-100 protein and CD34, but this feature is not particularly diagnostic. Histology is dominated by irregular spindle-shaped cells, locally invasive adipose and muscle, and the potential for recurrence and metastasis [7].

More kinase fusion sites have been reported, including *RET, RAF1, BRAF, ALK*, and others, due to the widespread use of next-generation sequencing (NGS) [8]. It has been observed that kinase-associated rearrangement spindle cell tumors have inhibitors that are associated with a favorable clinical prognosis [9, 10]. In this report, we present a case of S100-positive, CD34-negative spindle cell tumor with *MYH10-RET* fusion and provide a summary of the clinical features of the 6 cases of *MYH10-RET* that have been documented to date. A comprehensive understanding of *RET* rearrangement spindle cell tumor offers novel insights for the diagnosis and treatment of this neoplasm.

## 2. Materials and Methods

The present study was formally endorsed by the Institutional Review Board of Children's Hospital of Soochow University. The case was initiated in the Department of Pathology, Children's Hospital of Soochow University. A comprehensive review of all available hematoxylin and eosin-stained sections was conducted. Immunohistochemical studies were then performed on unstained sections provided by the referring pathologist using an automated immunohistochemical stainer (LEICA Biosystems, Germany). Antibodies used in the study included CD34 (QBEnd10, GM7165, Gene Tech.), S100 protein (GTM5, GT2286, Gene Tech.), Pan-TRK (RM423, ZA-0687, ZSGB-BIO), CD99 (EP8 GT2123, Gene Tech.), *α*-smooth muscle (*α*-SMA) (1A4, GM0851, Gene Tech.), Desmin (GTM2, GT2252, Gene Tech.), SOX-10 (GT2210, Gene Tech.), Myogenin (EP162, GM3559, Gene Tech.), Myo-D1 (EP212, GT2188, Gene Tech.), Bcl-2 (124, GM0887, Gene Tech.), HMB-45 (GM0634, Gene Tech.), SATB2 (GT2294, Gene Tech.), NKX2.2 (GT2345, Gene Tech.), AE1/AE3 (GM3515, Gene Tech.), BCOR (C-10, ZM-0029, ZSGB-BIO), ALK (4A4, GT2266, Gene Tech.), and Ki67 (GM027, Gene Tech.). All detection antibodies were accompanied by appropriate positive controls.

Fluorescence in situ hybridization (FISH) was performed on 3-*μ*m-thick unstained sections in a hybridisation instrument (TDH-500, ALLSHENG, Hangzhou, China) using *NTRK1/2/3* and *RET* two-color breakapart probes (Anbiping, Guangzhou, China) according to the protocol provided by the manufacturer. The analysis of FISH signals was conducted using a Nikon ECLIPSE Ni-L fluorescence microscope (Nikon Corporation, Japan). A total of 100 nuclei were evaluated per specimen. Cells were considered positive when at least 20% of the nuclei showed a split signal (defined as the distance between the orange and green signals being at least twice the estimated signal diameter).

RNA-seq-based NGS was performed on the Illumina NextSeq 6000 platform at Fudan University Shanghai Cancer Center (FUSCC). Total RNA was extracted from formalin-fixed, paraffin-embedded specimens (15 unstained sections, 4 *μ*m each), followed by cDNA synthesis. RNA sequence libraries were prepared with 20–100 ng of total RNA using the Trusight RNA Fusion Panel (Illumina, San Diego, California). Ultradeep panel sequences (632 genes) were analyzed for each case. Reads were independently compared to the human reference genome (hg19) using STAR (Version 2.3) and analyzed using STAR-Fusion.

The *MYH10 (E35)::RET (E12)(t(10; 17)(q11.21; p13.1))* fusion gene transcript sequence “TGGAGGTCAACATGCAGGCCATGAAGGCGCAGTTCGAGAGAGACCTGCAAACCAGGGATGAGCAGAATGAAGAGAAGAAGCGGCTGCTGATCAAACAG::GAGGATCCAAAGTGGGAATTCCCTCGGAAGAACTTGGTTCTTGGAAAAACTCTAGGAGAAGGCGAATTTGGAAAA” obtained by NGS sequencing was also validated by Sanger sequencing (Applied Biosystems 3500 Dx, Thermo Fisher Scientific Inc).

## 3. Results

### 3.1. Case Presentation

**Figure 1:**
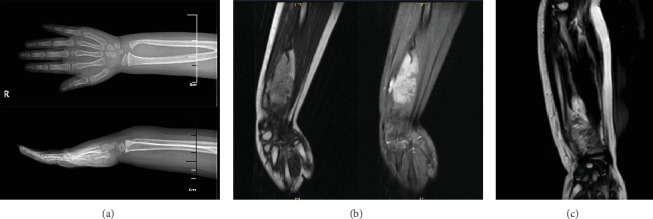
Radiologic examinations. (a) X-ray reveals abnormal bone quality of the right distal ulnar radius, characterized by roughness of the bone cortex and soft tissue swelling. (b) Magnetic resonance imaging revealed a T2 signal-enhancing mass in the right forearm with indistinct margins. (c) A magnetic resonance T2 image reveals tumor recurrence in muscle and the intermuscular space, accompanied by localized bone destruction.

The patient was a 3-year-old female with a neoplasm measuring 57 × 33 × 29 mm located on the right forearm. The patient exhibited clinical manifestations of swelling and discomfort, but no pain or history of trauma was reported. Laboratory tests revealed no abnormalities, and the patient's growth and development were within normal limits. A radiographic analysis revealed a lesion of a soft tissue mass between the right ulna and radius, with suspected periosteal destruction (Figure 1a). Magnetic resonance imaging revealed a T2 signal-enhancing mass in the right forearm, proximate to the wrist joint, between the ulnar radius, extending from the subcutaneous fat to within the deep muscularis propria (Figure 1b). The mass exhibited a poorly delineated margin, adjacent to the periosteum, and a well-defined bone marrow cavity. The presence of a congenital malformation tumor was considered, and it was determined to have a large extent of invasion. The tumor was completely resected. However, local recurrence of the tumor without distant metastasis was found at follow-up 8 months after surgical resection. The T2 magnetic resonance image shows tumor recurrence in the muscle and intermuscular space with local bone destruction (Figure 1c). The patient's father, mother, and brother were healthy; the patient's father had a history of lipoma, and there was no family history of genetic disorders (including multiple endocrine neoplasia Type 2 [MEN2]) or malignant tumors (including lung cancer and thyroid cancer).

### 3.2. Histopathologic and Molecular Analyses

**Figure 2:**
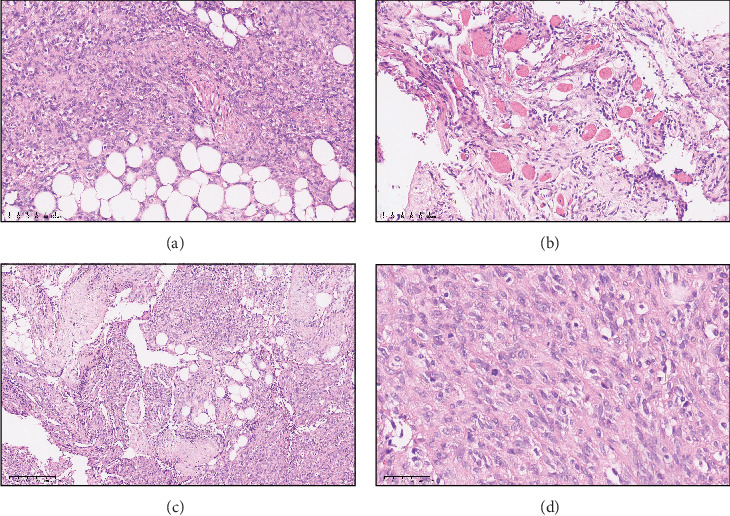
Morphology. (a) H&E showed tumor cells of moderate density that infiltrate adjacent adipose tissue, ×200. (b) Tumor involvement of surrounding muscle and collagen fibers. (c) Antler-shaped blood vessels, tumor cells grow around nerve fiber bundles, ×100. (d) Tumor cells are promiscuously arranged in fascicles; mitotic activity is visible, ×400.

Grossly, the excised sample was a grayish-yellow soft tissue with no visible envelope and no hemorrhage or calcification. Microscopically, the tumor cells exhibited a relatively uniform spindle-shaped morphology, characterized by a haphazard intertwined growth pattern (Figure 2a). The tumor cells manifested a dense packing pattern with sporadic pale eosinophilic cytoplasm, ovoid nuclei, and inconspicuous nucleoli. The cells infiltrated and proliferated within subcutaneous soft tissues and skeletal muscle, exhibiting characteristics consistent with lipofibromatosis (Figure 2b). In areas with reduced cellular density, reticulately arranged spindle-shaped cells were observed within a sparse, loosely myxohyaline stroma, accompanied by thin-walled dilated staghorn-shaped blood vessels, growing around the nerve fibers (Figure 2c). Mitotic figures were well identifiable (8/10 HPFs), and no significant vascular proliferation or neoplastic osteogenesis was observed (Figure 2d).

**Figure 3:**
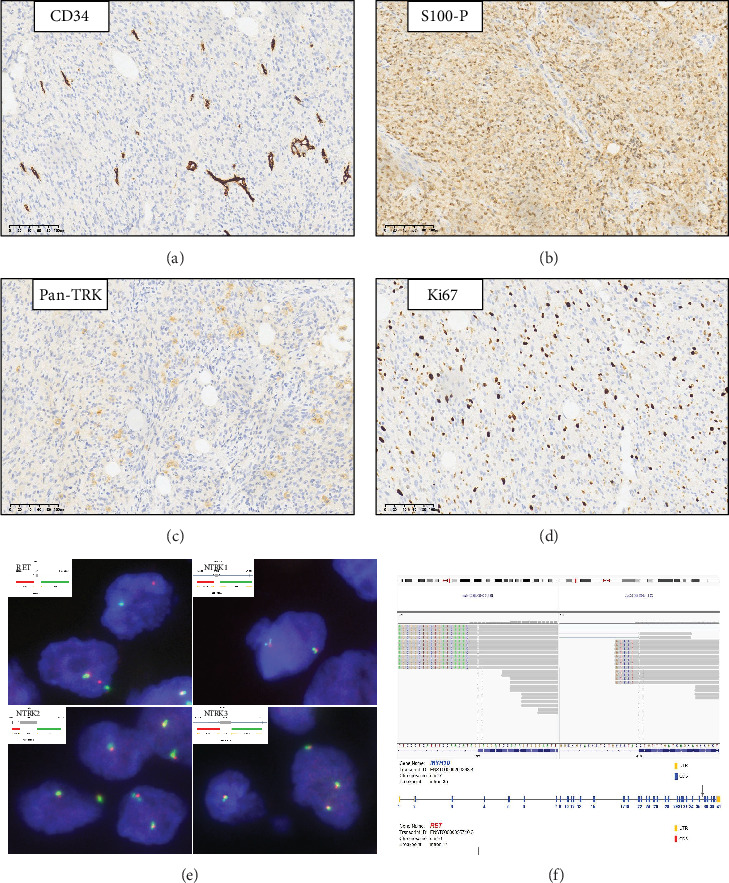
Immunohistochemistry and molecular characterization. (a) Negative staining of CD34. (b) S100 protein showing diffuse staining. (c) Weak expression of pan-tyrosine receptor kinase (Pan-TRK). (d) Ki67 index of approximately 20%, × 100. (e) RET, NTKR1/2/3 rearrangement tested by FISH analysis. (f) Integrative Genomics Viewer (IGV) showing *MYH10::RET* fusion.

Spindle tumor cells do not express CD34, a notable distinction from the surrounding vessels (Figure 3a), and they also exhibit diffuse and intense S100 protein expression (Figure 3b). Pan-TRK staining revealed focal, weak, and punctate cytoplasmic and nuclear expression (Figure 3c). Conventional analytical markers, including *α*-SMA, Desmin, SOX-10, Myogenin, Myo-D1, Bcl-2, HMB-45, AE1/AE3, BCOR, and ALK, were not detected. However, CD99 exhibited diffuse positive expression. Immunostaining for H3K27Me3 was preserved, accompanied by a Ki67 index of approximately 20% (Figure 3d). The tumor first underwent an intraoperative frozen section, which diagnosed it as a spindle cell tumor. The paraffin specimen was initially diagnosed as a low-grade malignant spindle cell tumor.

FISH analysis identified the presence of RET gene rearrangements, but no alterations in the *NTRK1/2/3* genes were detected (Figure 3). Subsequent RNA-seq-based NGS analysis revealed a *MYH10-RET* fusion, with breakpoints involving exon 35 of *MYH10* and exon 12 of the *RET* gene (*MYH10 exon 35::RET exon 12* fusion) (Figure 3). The final pathological diagnosis was RET-rearranged spindle cell neoplasm, determined by combining the results of histopathology, NGS, and FISH tests.

## 4. Discussion

RET rearranged spindle cell tumors represent a rare form of soft tissue neoplasm, the current classification of which is as a type of *NTRK* and its related kinase fusion spindle cell tumors, in addition to *RAF1, BRAF, ALK* genes, and others. Among these, *NTRK* rearranged spindle cell neoplasm has received the most extensive study. The histological hallmark of this tumor group is an irregular arrangement of spindle cells, accompanied by local invasiveness and the potential for local recurrence and metastasis. However, it is generally accepted that positive expression of CD34 and S100, suggestive of *NTRK* and its related kinase fusion spindle cell tumors, does not have specialized IHC to aid in the diagnosis of such spindle cell tumors, thus complicating the process of accurate diagnosis. The advent of NGS assays has led to the recent identification of kinase-fused spindle cell tumors in soft tissue tumors. Some of the kinases (*NTRK, RET*, etc.) involved in these tumors have been identified as altered, paving the way for targeted therapy with larotrectinib and entrectinib. However, further clinical investigation is necessary to ascertain the specific efficacy of these drugs in spindle cell tumors.

Neurotrophic tropomyosin-receptor kinase (Trk) is a family of transmembrane tyrosine kinases, which includes three transmembrane neurotrophin receptors: TrkA, TrkB, and TrkC. The genes that encode these receptors are *NTRK1, NTRK2,* and *NTRK3*, respectively [11]. The histological spectrum of *NTRK*-rearranged spindle cell tumors (excluding IFS) is broad. These tumors range from low to medium-grade morphologically uniform LPF-NTs to high-grade MPNSTs, and may exhibit increased mitotic activity and local necrosis. The genetic alterations involving *NTRK* are characterized by the presence of specific chaperone genes, including *ETV6, STRN, EML4,* and *LMNA*, among others [12, 13]. *NTRK* rearrangements most commonly involve the *NTRK1* gene translocation, followed by the *NTRK3* gene, and to a lesser extent the *NTRK2* gene [6]. In light of the potential for local recurrence or distant metastasis of this tumor and the advent of tyrosine kinase inhibitors (TKIs) targeted therapy, it is imperative to consider these treatment modalities for this subtype of soft tissue tumor with specific gene mutations.

*RET* is a receptor tyrosine kinase proto-oncogene located on chromosome 10q11.2. Like *NTRK*, *RET* activation triggers the *RAS-RAF-MAPK* cascade. This cascade promotes cell survival and proliferation. The *NTRK, RAF1,* and *BRAF* genes are all members of this signaling pathway [14]. In this study, RNA sequencing identified a *MYH10::RET* fusion. The *RET* breakpoint is located in exon 12 and, similar to reported fusion transcripts, retains the tyrosine kinase structural domain. Its chaperone, the NMII heavy chain-encoding IIB (*MYH10*) gene, is an essential component of the cytoskeleton that is expressed in a variety of cell types. In neoplastic tissues, *MYH10* functions as an oncogene, with increased expression in various types of cancer, including breast cancer, gliomas, and meningiomas [15, 16]. This study presents a case of *MYH10 exon 35::RET exon 12* fusion in a *RET*-rearranged spindle cell tumor (LPF-NT) that has not been previously documented. The literature has documented additional *RET* fusion partners, including *PRKAR1A*, *SPECC1L*, *CCDC6*, *CLIP2*, *KIF5B*, *KIAA1217*, *KHDRBS1*, *NCOA4*, *VCL*, and *TFG* [7, 17–21].

*MYH10-RET* fusions represent the most prevalent genetic alteration in *RET*-rearranged spindle cell tumors. In this study, we conducted a retrospective analysis of the clinical features associated with six cases of spindle cell tumors harboring *MYH10-RET* rearrangements (Table 1). These tumors were found to predominantly occur in younger children, approximately 3 years of age (excluding a 47-year patient). The tumor site is not specific, and this is the first report of *RET* rearrangement tumors occurring in the forearm. The histologic pattern was predominantly IFS-like, with a favorable overall prognosis. Immunohistochemical analysis revealed that most *MYH10-RET* and *NTRK*-rearranged spindle cell tumors were positive for S100 and CD34, but this did not aid in diagnosis. Notably, we also identified a case with rare CD34-negativity. Initially, when *NTRK*-rearranged spindle cell tumors were described, it was widely accepted that tumor cells were S100 and CD34-positive. However, recent reports suggest that such kinase fusion spindle cell tumors can be CD34-negative or even S100-negative or focally positive, indicating the heterogeneity of tumor differentiation and protein expression in this spindle cell type [5]. These tumors exhibit a broad morphologic spectrum, relatively nonspecific immunologic features, and a wide range of clinical profiles and behaviors, making them difficult to diagnose by conventional pathology, and NGS-based molecular testing is extremely important.

Although selpercatinib is a targeted therapy for RET in the treatment of medullary thyroid cancer and non-small cell lung cancer, it is not indicated for RET-rearranged spindle cell tumors or children under the age of 4 in mainland China [22]. To avoid the side effects of targeted therapy, this case underwent complete surgical resection without prior RET-targeted therapy. The patient was followed up for 18 months postsurgery. Local recurrence occurred at 8 months postsurgery, but there was no significant mass effect, only visible on imaging, and no distant metastasis was present. The patient's other conditions remained normal. Due to the imaging suggestive of periosteal reaction, CD99, SATB2, and NKX2.2 staining was performed to exclude osteosarcoma and Ewing sarcoma. *RET*-rearranged spindle cell tumors were found to express CD99, suggesting that the tumor cells had more primitive features. In another case of spindle cell tumor with *TFG-RET* fusion, the cytoplasm of CD99 was also positive but not characteristically diagnostic [23]. In the present case, the tumor cells were negative for CD34 and focally positive for Pan-TRK, which is different from previous knowledge and represents the first such finding in *RET*-rearranged spindle cell tumors.

In this case, the tumor was large (57 mm in diameter) and imaging suggested possible bone tissue invasion. The tumor's boundaries were indistinct, extending into the surrounding fat, muscle, and nerve tissues. The spindle-shaped tumor cells exhibited slight heterogeneity with visible nuclear pleomorphism and locally showed high proliferative activity. Despite undergoing complete resection and the absence of targeted therapy, the tumor recurred after 8 months. Consequently, the presence of the following morphological features may indicate *RET*-rearranged or other related kinase-rearranged spindle cell tumors with more malignant biological characteristics: tumor diameter > 50 mm, permeative borders, tumor cell atypia, ≥ 10 mitoses per 10 HPF, and negative expression of CD34 and/or S100.

However, in contrast to IFSs, the biology of NTRK and other related kinase-arranged spindle cell tumors occurring in children exhibits an intermediate biological behavior. To ensure the most accurate and comprehensive assessment possible, a range of metrics should be employed. In summary, this study describes the clinicopathological characteristics and molecular phenotype of a *RET*-rearranged spindle cell tumor with a *MYH10::RET* fusion. The study highlights the importance of genome sequencing in identifying spindle cell tumors associated with tyrosine kinase rearrangements, particularly the rare *RET*-rearranged spindle cell tumors, which manifest nonspecific morphology and immunophenotype.

## Figures and Tables

**Table 1:** Clinicopathologic and molecular features of *MYH10-RET* rearranged spindle cell tumors.

**No.**	**Age (years)/sex**	**Location**	**Size (cm)**	**Diagnosis**	**IHC**	**MYH10 broken sites**	**Outcomes and follow-up (months)**
Current case							
1	3/F	Right limb	5.7	LPF-NT	CD34-, S100-P+, CD99 +	Exon 35	Tumor recurrence (8)
Previously published cases							
2	0/F	Lumbosacral	NA	Infantile myofibromatosis	SMA focal +, CD34+	NA	AWD (10)
3	47/M	Buttock	11	High-grade-MPNST-like	S100-P focal +, CD34+	Exon 37	Lung metastases, DOD (24)
4	6 months/F	Pelvis	13	Infantile FS-like	S100-P diffuse +, CD34+	NA	AWD (6)
5	1/F	Pelvis	10.3	Infantile FS-like	CD34+, S100-P+, SMA focal+	NA	Good response to RET inhibitor, AWD (16)
6	11/M	Hand	4.5	Infantile FS-like	SMA, CD34 focal+	NA	NED (12)
7	7 months/F	Paraspinal and retroperitoneal	NA	Infantile myofibroma	NA	NA	Lung metastases, good response to selpercatinib, NA

Abbreviations: AWD, alive with disease; F, female; FS, fibrosarcoma; IHC, immunohistochemistry; LPF-NT, lipofibromatosis-like neural tumor; M, male; MPNST, malignant peripheral nerve sheath tumor; NA, not available; NED, no evidence of disease; SMA, smooth muscle actin.

## Data Availability

The data used to support the findings of this study are included within the article.
